# gesp: A computer program for modelling genetic effective population size, inbreeding and divergence in substructured populations

**DOI:** 10.1111/1755-0998.12673

**Published:** 2017-04-21

**Authors:** Fredrik Olsson, Linda Laikre, Ola Hössjer, Nils Ryman

**Affiliations:** ^1^ Department of Mathematics Division of Mathematical Statistics Stockholm University Stockholm Sweden; ^2^ Department of Zoology Division of Population Genetics Stockholm University Stockholm Sweden; ^3^ Present address: Department for Development of Processes and Methods Statistics Sweden Stockholm Sweden

**Keywords:** eigenvalue effective size, inbreeding coefficient, inbreeding effective size, kinship coefficient, metapopulation effective size, migration, software, subpopulation differentiation

## Abstract

The genetically effective population size (*N*_*e*_) is of key importance for quantifying rates of inbreeding and genetic drift and is often used in conservation management to set targets for genetic viability. The concept was developed for single, isolated populations and the mathematical means for analysing the expected *N*_*e*_ in complex, subdivided populations have previously not been available. We recently developed such analytical theory and central parts of that work have now been incorporated into a freely available software tool presented here. gesp (Genetic Effective population size, inbreeding and divergence in Substructured Populations) is R‐based and designed to model short‐ and long‐term patterns of genetic differentiation and effective population size of subdivided populations. The algorithms performed by gesp allow exact computation of global and local inbreeding and eigenvalue effective population size, predictions of genetic divergence among populations (*G*_*ST*_) as well as departures from random mating (*F*_*IS*_, *F*_*IT*_) while varying (i) subpopulation census and effective size, separately or including trend of the global population size, (ii) rate and direction of migration between all pairs of subpopulations, (iii) degree of relatedness and divergence among subpopulations, (iv) ploidy (haploid or diploid) and (v) degree of selfing. Here, we describe gesp and exemplify its use in conservation genetics modelling.

## 1 Introduction

Assessing and monitoring the expected rate of loss of genetic variation and the degree of population differentiation is of key importance in molecular ecology and conservation genetics. It is therefore important to quantify inbreeding within individuals and kinship coefficients between them as a function of time. One fundamental parameter in this respect is the genetically effective population size *N*_*e*_ (Wright, 1931, 1938), defined as the size of an ideal population exhibiting the same rate of increased inbreeding as the nonideal population under study. *N*_*e*_ is a key tool in conservation genetics, but guidelines are based on models referring to a single, isolated population of constant size (Allendorf & Ryman, 2002; Franklin, 1980; Soulé, 1986; Traill, Brook, Frankham, & Bradshaw, 2010). This is primarily because much less is known about the behaviour of *N*_*e*_ in substructured populations (i.e. metapopulation‐*N*_*e*_) as compared to isolated homogeneous ones (Wang & Caballero, 1999; Waples, 2010). This is in spite of a long history of theoretical developments for *N*_*e*_ of substructured populations that includes effects of symmetrical migration between subpopulations of the island model (Wright, 1951) and stepping stone migration models (Kimura, 1953; Weiss & Kimura, 1965), impact of strong migration (Nagylaki, 1980) and relationships between different types of *N*_*e*_ (Whitlock & Barton, 1997).

The coefficient of gene differentiation *G*_*ST*_ of Nei (1973) is another essential parameter that quantifies the proportion of genetic variation due to genetic differences between subpopulations. It extends the fixation index *F*_*ST*_ (Wright, 1943, 1951) to multiallelic and multilocus situations. In some applications, it is also important to quantify the degree of nonrandom mating in terms of departures from Hardy–Weinberg proportions, either within subpopulations (*F*_*IS*_) or within the total population (*F*_*IT*_).

We have recently developed a unified mathematical framework for haploid and diploid structured populations that can be used to compute expected inbreeding and kinship coefficients, effective population size, genetic divergence and departures from random mating in populations that consist of various numbers of more or less interconnected subpopulations whose size may vary over space and time (Hössjer, Laikre, & Ryman, 2016; Hössjer, Olsson, Laikre, & Ryman, 2014, 2015). This newly developed theory allows computation and modelling of parameters of complex metapopulations that has previously not been possible. It is applicable to selectively neutral inheritance at Y‐chromosomes, mitochondrial DNA (haploid populations) and autosomes (diploid populations).

Here, we present a computer program gesp (Genetic Effective population size, inbreeding and divergence in Substructured Populations) that performs several of the analytical computations outlined in Hössjer et al. (2014, 2015). gesp can be used to model exact local and global rates of inbreeding, haploid and diploid inbreeding as well as eigenvalue effective size, and population divergence in a substructured population. gesp focuses on geographic subdivision and ignores other types of structure such as overlapping generations.

Several software exist for addressing various issues relating to genetically effective population size, and they can be classified into the following three categories: computer programs that (i) simulate drift or other processes and produce data that can be used for estimating *N*_*e*_, (ii) estimate *N*_*e*_ from empirical data and (iii) compute predictions of *N*_*e*_ from demographic parameters by an exact algorithm. Examples of category 1 software include simulation programs popsim (Hampe, Wienker, Schreiber, & Nürnberg, 1998), vortex (Lacey, 2000) and easypop (Balloux, 2001). tempofs (Jorde & Ryman, 2007), ldne (Waples & Do, 2008), onesamp (Tallmon, Koyuk, Luikart, & Beaumont, 2008), gone (Coombs, Letcher, & Nislow, 2012) and neestimator (Do et al., 2014) represent the second category of programs that estimate *N*_*e*_ from empirical genotype or allele frequency data. Category 3 includes gesp and other programs, such as agene (Waples, Do, & Chopelet, 2011), that iteratively compute a forward prediction of *N*_*e*_. However, in contrast to gesp, agene focuses on a single, isolated population with age structure. gesp complements agene and other available software for *N*_*e*_‐modelling by performing exact calculations for spatially substructured populations using theory that has not previously been available. No other program currently performs such computations.

gesp can be used to model, for example metapopulation‐*N*_*e*_ and such modelling can use simulated, empirical or hypothetical estimates of local effective size, inbreeding levels and rates of migration as input. This can aid in avoiding incorrect conservation management recommendations for *N*_*e*_ of substructured populations as have been reported (Holley et al., 2014). For instance, we have recently applied GESP to the case study of the Fennoscandian wolf metapopulation to address questions of how large local effective sizes and what rates of gene flow that are needed to reach conservation genetic goals (Laikre, Olsson, Jansson, Hössjer, & Ryman, 2016).

Below we describe gesp, its parameters, and exemplify how the program can be used to aid researchers of molecular ecology and conservation genetics to explore the impact that various future demographic scenarios may have on effective size, inbreeding and subpopulation differentiation.

## 2 What gesp Does

In this section, we briefly introduce notation and describe the mathematics behind gesp. The metapopulation is assumed to have *s* subpopulations. The theory allows *s* to change over time (Hössjer et al., 2014, 2015), but in the current implementation of gesp, it is constant. On the other hand, the sizes of all subpopulations or the migration rates between them can differ and vary over time. We consider a selectively neutral and polymorphic locus and study how the genetic composition of the population at this locus is expected to evolve over discrete time steps *t *=* *0, 1, …, *t*_max_, typically generations. Here, *t *=* *0 represents the present, *t *>* *0 the future and *t*_max_ + 1 is the number of time points.

### 2.1 Identity‐by‐descent parameters and their recursions

Let A be the set of all types of gene pairs, that is pairs of alleles. The most crucial building block of gesp is a number of probabilities

(1) $$f_{ta}=1-h_{ta}$$

that a gene pair of type *a* ∈ A is identical by descent IBD (or identical by state, IBS) if drawn randomly from the population at time *t*. For a haploid population, where each individual has a single gene copy, there are *d *=* s* ^2^ different types *a *=* ij* that specify the ordered pair *i* and *j* of subpopulations to which the two genes belong. We then refer to *f*_*tij*_ as an average inbreeding coefficient between subpopulations *i* and *j* at time *t*. For a diploid population, each individual carries two homologous genes. Whenever the two genes are drawn from the same subpopulation *i*, we must distinguish whether they belong to the same (*a *=* i*) or to different (*a *=* ii*) individuals. This gives a total of *d *=* s* ^2^ + *s* diploid gene pair types. The corresponding quantities in Equation (1) are referred to as average inbreeding coefficients (*a *=* i*), average kinship coefficients within subpopulations (*a *=* ii*) or average kinship coefficients between subpopulations (*a *=* ij*,* i *≠ *j*).

An important aspect of gesp is to use matrix analytic methods to describe time progression of the inbreeding and kinship coefficients, with genetic drift, migration and mutation as the three forces of genetic change. In the haploid as well as the diploid case, this is achieved by gathering all non‐IBD probabilities at time *t* into a column vector ***h***_*t*_ **=* *(*h*_*ta*_, a ∈ A)′ of length *d*. If the two genes are drawn without replacement, the vector of non‐IBD probabilities obeys a linear recursion

(2) $$h_{t}={\left(1-\mu \right)}^{2}D_{t}h_{t-1}+\left[1-{\left(1-\mu \right)}^{2}\right]1$$

between time points *t *− 1 and *t*, where ***D***_*t*_ is a square matrix of order *d*,** 1** is a column vector of *d* ones and μ is the probability that a gamete mutates under an infinite alleles model (Kimura, 1971). A recursion similar to Equation (2) holds if the two genes are drawn with replacement.

For haploid and diploid models, the elements of ***D***_*t*_ are functions of the local census and effective sizes *N*_*ti*_ and *N*_*eti*_ of all subpopulations *i* at time *t*, as well as the migration rates *m*_*tji*_ from one subpopulation *j* to another subpopulation *i* between time points *t *− 1 and *t*. As migration is specified forward in time, from one time point to the next, we refer to *m*_*tji*_ as a forward migration rate. Some additional parameters are needed for diploid models, because reproduction may occur either by selfing or crossing. This requires specifying the rate of selfing as well as whether mating occurs before or after migration.

### 2.2 Subpopulation weights

When computing, for example the effective population size for the metapopulation as a whole, the contribution from the different subpopulations must be weighted, and this can be done in several ways regarding the weights of separate subpopulations. The predefined weighting schemes in gesp include that the subpopulation weights are either uniform (i.e. the same for all subpopulations), proportional to size, proportional to reproductive value (i.e., populations that contribute more to the system as a whole because of migration rates and patterns are given more weight; cf. Fisher, 1958; Felsenstein, 1971) or allocated to particular subpopulations so that other subpopulations are ignored by receiving zero weights. Which of these weights to use depends on the goals of the investigator. Size proportional weights treat all individuals of the metapopulation equally, reproductive weights corresponds to the long‐term behaviour of the system, and local weights focus on one particular subpopulation. Further, it is possible to define user‐specified subpopulation weights as any non‐negative numbers *w*_1_, …, *w*_*s*_ that sum to one ($\sum_{i=1}^{s}w_{i}=1$). A weighting scheme is global if at least two *w*_*i*_ are positive, whereas it is local if one subpopulation *i* receives full weight (*w*_*i*_ **=* *1). It is convenient to interpret all *w*_*i*_ as probabilities of sampling genes from the various subpopulations, because this naturally defines weights *W*_*a*_ for all *a* in terms of probabilities of sampling gene pairs of type *a*. This can be done in different ways, and we distinguish between a number of different sampling schemes for gene pairs. In the diploid case, the three most important schemes are *T*,* S* and *I*. They differ as to whether the two genes are chosen independently from the total (*T*) population (weights *W*_*a*_ **=* W*_*Ta*_), from the same randomly chosen subpopulation (*S*; weights *W*_*a*_ **=* W*_*Sa*_) or from the same randomly chosen individual (*I*; weights *W*_*a*_ **=* W*_*Ia*_). Given that subpopulation weights have been specified, the probability is

(3) $$\begin{array}{cc} h_{Tt} & =\sum_{a\in A}W_{Ta}h_{ta}=1-f_{Tt}\\ h_{St} & =\sum_{a\in A}W_{Sa}h_{ta}=1-f_{St}\\ h_{It} & =\sum_{a\in A}W_{Ia}h_{ta}=1-f_{It} \end{array}$$

that two genes are not IBD, if sampled at time *t* by any of the three schemes *T*,* S* or *I*. If the gene pair is sampled without replacement, the formulas for *h*_*Tt*_ and *h*_*St*_ are the same, whereas the definition of *h*_*It*_ is multiplied by a factor of two.

In the diploid case, *f*_*It*_ is a weighted average of the inbreeding coefficients *f*_*ti*_ of all subpopulations *i* at time *t* with positive weights, whereas *f*_*St*_ is a weighted average of the inbreeding coefficients *f*_*ti*_ *and* kinship coefficients *f*_*tii*_ within subpopulations, for all subpopulations *i* with positive weights.

### 2.3 Notions of effective population size in gesp

Some definitions of effective size incorporate mutations (Ewens, 1989; Maruyama & Kimura, 1980). This may be of interest for long‐time scenarios, and the theory in Hössjer et al. (2014, 2015) includes the effect of mutations. However, in the present implementation of gesp, we follow the most common approach and assume there are no mutations (μ* *=* *0), or equivalently, pay no attention to mutations in the definition of non‐IBD probabilities in Equation (4). These probabilities are used to compute a number of different effective sizes over different time horizons [*t*,* t* + τ]. In the diploid case, the inbreeding effective size

(4) $$N_{eI}\left(\left[t,t+\tau \right]\right)=\frac{1}{2\left[1-{\left(h_{I,t+\tau }/h_{It}\right)}^{1/\tau }\right]}$$

quantifies the average rate at which the non‐IBD probabilities of a gene pair decreases between *t* and *t* + τ, when sampled without replacement according to scheme *I* at both time points. Global and local inbreeding effective sizes differ as to whether global and local subpopulation weights *w*_*i*_ are used. In order to compute Equation (5), it is necessary to define a scenario for how the demography of the population evolves during [*t*,* t* + τ], and *h*_*It*_ requires knowledge of kinship and inbreeding coefficients at time *t*. The latter can be chosen arbitrarily from simulated or real data. In particular, it is not required that the population is in equilibrium at time *t*.

gesp provides inbreeding effective size for the global metapopulation and for separate subpopulations over time intervals, either from the start *t *=* *0 to another specified point *t*, or from one time point *t* to the next *t *+* *1. The latter rate of inbreeding from one generation to the next corresponds to an *instantaneous effective size*. We have previously suggested the term *realized effective size* (*N*_*eR*_) for an instantaneous effective size that is determined both by genetic drift and migration (Laikre et al., 2016). If subpopulation *i* receives full weight (*w*_*i*_ **=* *1), then *N*_*eRti*_ **=* N*_*eI*_([*t*,* t *+* *1]) is the realized effective size of *i* at time *t*. This is a local inbreeding effective size that equals *N*_*eti*_ if *i* is isolated. But in general, the two quantities differ, as the local effective size *N*_*eti*_ is an input parameter of the model that is only affected by genetic drift within *i* between time points *t* and *t *+* *1, whereas *N*_*eRti*_ is an output parameter that is also influenced by immigration into *i* from the other subpopulations between *t* and *t *+* *1. More generally, *N*_*eI*_([*t*,* t* + τ]) quantifies the average combined impact of genetic drift and migration for time intervals of any length τ, for those subpopulations that are part of the weighting scheme. For management applications, we argue that *N*_*eI*_ is a more relevant concept than those notions of *N*_*e*_ that only include genetic drift, as the effects of migration and drift are hard to separate (and estimate), in particular when the subpopulation structure is cryptic and partly unknown.

The eigenvalue effective population size *N*_*eE*_ gives the long‐term equilibrium rate at which inbreeding increases (τ → ∞). This requires some additional assumptions, such as time‐invariant migration rates and subpopulation sizes, and that no group of subpopulations is isolated, to make ***D***_*t*_ **=* **D*** in Equation (2) time invariant with a unique largest eigenvalue λ_max_ (***D***). The inbreeding effective population size then has the long‐term limit

$$\lim_{\tau \to \infty }N_{eI}\left(\left[t,t+\tau \right]\right)=N_{eE}=\frac{1}{2[1-\lambda_{\text{max}}\left(D\right)]}$$

For many types of migration schemes, neither the instantaneous nor the long term *N*_*e*_ gives the full picture. Rather, the whole curve τ → *N*_*e*_([*t*,* t* + τ]) is needed to capture the rate at which inbreeding increases in a subdivided population.

### 2.4 Fixation indices

In order to quantify subpopulation differentiation and departures from random mating, we use

(5) $$\begin{array}{cc} g_{STt} & =\left(h_{Tt}-h_{St}\right)/h_{Tt}\\ f_{ISt} & =\left(h_{St}-h_{It}\right)/h_{St}\\ f_{ITt} & =\left(h_{Tt}-h_{It}\right)/h_{Tt} \end{array}$$

to predict the fixation indices *G*_*STt*_, *F*_*ISt*_ and *F*_*ITt*_ at time *t*. The quantities on the right‐hand sides of Equation (5) are all defined in Equation (4) under the assumption that pairs of genes are drawn with replacement. The predicted coefficient of gene differentiation is only applicable for global subpopulation weights, and it satisfies 0 ≤ *g*_*STt*_ ≤ 1, with the lower and upper bounds attained when subpopulations are genetically identical or fully diverged. The other two fixation indices satisfy  − 1 ≤ *f*_*ISt*_, *f*_*ITt*_ ≤ 1. A necessary condition for attaining the lower and upper bounds is that all or no individuals have heterozygous genotypes. Random mating and selfing give *f*_*ISt*_ a value close to 0, with a small negative bias caused by a Levene effect (Crow & Kimura, 1970).

## 3 Parameters in gesp

In gesp, all input parameters are specified using the graphical user interface. Table 1 contains a summary of some of the most important quantities used by the program. The output of gesp is shown in the interface. Figures can be saved in various formats, and all results can be exported to a csv‐file. All input and output parameters are described in detail in the manual (Olsson, 2017).

**Table 1:** Population genetic parameters used by gesp. They all apply to a diploid model. Some quantities are slightly different for haploid models, see the reference manual (Olsson, 2017) for details

Symbol	Definition
*s*	Number of subpopulations
*t*	Discrete time point (typically a generation number)
*t*_max_	Number of time points after *t *=* *0
*N*_*ti*_	Local census size of subpopulation *i* at time *t*
*N*_*eti*_	Local effective size of subpopulation *i* at time *t* under isolation
*m*_*tji*_	Forward migration rate from subpopulation *j* to subpopulation *i* between time points *t *− 1 and *t*
μ	Mutation probability per gamete
A	Set of all types of gene pairs
*d*	Number of possible gene pairs
*f*_*ti*_	Inbreeding coefficient of individuals of subpopulation *i* at time *t*
*f*_*tij*_	Kinship or coancestry coefficient of two individuals from subpopulations *i* and *j* at time *t*
*f*_*It*_	Average inbreeding coefficient within individuals at time *t*
*f*_*St*_	Average inbreeding/coancestry coefficient within subpopulations at time *t*
*f*_*Tt*_	Average inbreeding/coancestry coefficient in the total population at time *t*
*h*_*ti*_	=1 − *f*_*ti*_
*h*_*tij*_	=1 − *f*_*tij*_
*h*_*It*_	=1 − *f*_*tI*_
*h*_*St*_	=1 − *f*_*tS*_
*h*_*Tt*_	=1 − *f*_*tT*_
τ	Length of time interval of genetic drift
*N*_*eI*_([*t*,* t* + τ])	Inbreeding effective size over time interval [*t*,* t* + τ]
*N*_*eI*_([*t*,* t *+* *1])	Instantaneous inbreeding effective size over one single generation at time *t*
*N*_*eRti*_	Realized effective size of subpopulation *i* at time *t*. It is a special case of instantaneous effective size when subpopulation *i* receives full weight. It includes the effect of genetic drift within *i* and migration into *i*
*N*_*eE*_	Eigenvalue effective size
*G*_*STt*_	Coefficient of gene differentiation at time *t*
*g*_*STt*_	Prediction of *G*_*STt*_
*F*_*ISt*_	Fixation index of individuals within subpopulations, time *t*
*f*_*ISt*_	Prediction of *F*_*ISt*_
*F*_*ITt*_	Fixation index of individuals within the total population, time *t*
*f*_*ITt*_	Prediction of *F*_*ITt*_

## 4 gesp in Conservation Genetic Modelling

One of the main purposes of gesp is to analyse how inbreeding dynamics and effective population sizes are affected by various migration scenarios, including populations with varying subpopulation sizes and local bottlenecks. Even though the number of subpopulations is kept fixed, it is still possible to put some local census sizes to zero and thereby incorporate subpopulation extinction and recolonization. Here, we describe an example population in which one of the subpopulations exhibits a local bottleneck, although not a complete extinction. The example is further described in the manual (Olsson, 2017), where the model is specified with a step‐by‐step instruction. See also Laikre et al. (2016) for a case study of the Fennoscandian wolves where the theory that has been incorporated in gesp is used for practical conservation genetic modelling, including suggestions of general conservation genetic targets for metapopulations (the publication is available for download at the gesp website).

**Figure 1:**
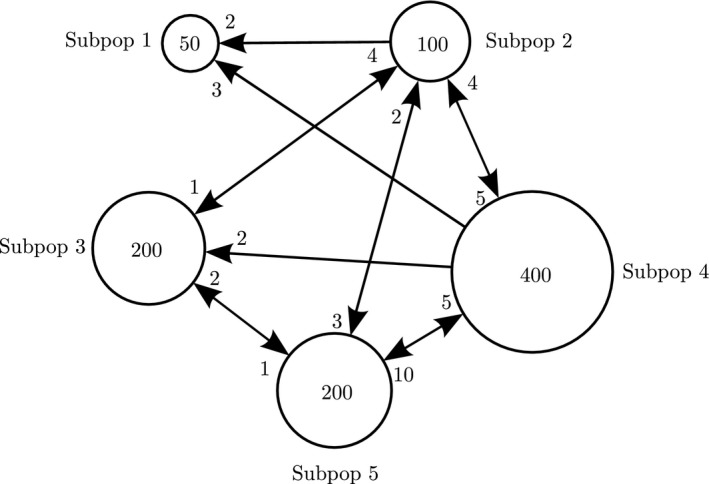
Schematic overview of a population divided into five subpopulations. All local census and local effective population sizes are the same, given as numbers inside the circles. The integer at each arrow refers to the number of migrants per generation between this pair of subpopulations

**Figure 2:**
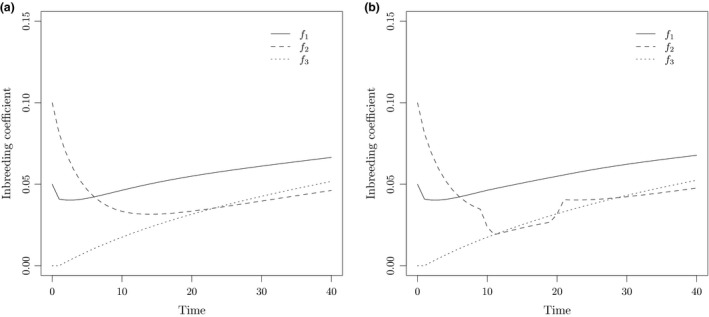
Inbreeding coefficients for subpopulations 1–3 of Figure 1. In the left subplot (a) the subpopulation sizes are constant, whereas in the right subplot (b), the size of subpopulation 2 has been reduced from 100 to 30 between generations 10 and 20 in order to model a local bottleneck see Figure 1

Consider a diploid population with no selfing divided into five subpopulations with migration scheme and subpopulation sizes described in Figure 1. Let the initial inbreeding and kinship coefficients be 0.05 for subpopulations 1 and 4, 0.1 for subpopulation 2 and 0 for subpopulations 3 and 5. Starting levels of kinship between subpopulation pairs is zero for all pairs. The time dynamics of the inbreeding coefficients for the first 40 generations are shown in Figure 2a), for subpopulations 1, 2 and 3. Now, we change the size of subpopulation 2 to 30 between generations 10 and 20. This is done by keeping all migration rates fixed, but the number of nonmigrants in subpopulation 2 is reduced from 90 to 20. Figure 2b) displays the effect of this local bottleneck on the inbreeding coefficients for subpopulations 1, 2 and 3.

To summarize, with gesp, it is possible to model a substructured population with a general migration scheme and compute analytical values of, for example, local and global rates of inbreeding, effective population sizes and population divergence.

## 5 Potential for Future Extension

At this point, only parts of the Hössjer et al. (2014, 2015, 2016) mathematical framework for modelling various genetic aspects of substructured populations have been implemented in gesp. Thus, there is potential for further extensions of gesp, that is, to combine geographic structure with overlapping generations, as outlined in Hössjer et al. (2015). Further, we believe it is possible to extend the theory to X‐chromosomes, by generalizing results in Nagylaki (1995) for isolated populations to those with geographic subdivision.

## 6 Download and Usage

The program, together with its manual, can be downloaded from the website www.zoologi.su.se/research/GESP. The manual (Olsson, 2017) covers information about the installation process, a detailed overview of the interface and a number of examples.

## Author Contributions

All the authors wrote the manuscript jointly and participated in designing and testing of the program. F.O. developed the program.
